# Special Issue “Molecular and Cellular Research in Pregnancy-Related Complications”

**DOI:** 10.3390/ijms27041734

**Published:** 2026-02-11

**Authors:** Regina Komsa-Penkova

**Affiliations:** 1 Department of Biochemistry, Medical University Pleven, 1, Sv. Kliment Ohridski Str., 5800 Pleven, Bulgaria; rkomsa@gmail.com; 2 Leonardo da Vinci Center of Competence in Personalized Medicine, 3D and Telemedicine, Robotic and Minimally Invasive Surgery, 1, Sv. Kliment Ohridski Str, 5800 Pleven, Bulgaria

## 1. Multifaceted Challenge of Maternal Health

Maternal reproductive complications represent one of the most pressing challenges in modern obstetrics, affecting millions of pregnancies worldwide and carrying profound implications for both maternal and offspring health [1]. Recent advances in molecular biology, omics technologies [2,3], and systems level understanding have begun to illuminate the intricate pathophysiological mechanisms of conditions such as gestational diabetes mellitus (GDM) [4], preeclampsia (PE) [5], recurrent pregnancy loss (RPL), and other disorders in pregnancy. The paradigm shift in our understanding emphasizes the critical role of placental dysfunction as a central hub from which multiple pathological cascades originate.

This editorial synthesizes recent research that collectively reveals pregnancy complications as complex connected phenomena/pathology involving multiple organ systems, genetic predispositions, and molecular pathways rather than isolated pathological events.

## 2. Genetic and Molecular Foundations of Pregnancy Complications

The application of comprehensive omics approaches to RPL has unveiled an extraordinary complexity of molecular perturbations spanning genomics, epigenetics, transcriptomics, metabolomics, and proteomics [6,7]. The identification of 14 candidate genetic variants through whole exome sequencing, including mutations in TLE6, NLRP7, and FSHR, provides important insights into early embryonic development failures [8]. Comparable in significance is the discovery of dysregulated microRNAs (miR-125a, miR-146a, miR-25, miR-32, miR-222) and long non-coding RNAs, which collectively coordinate networks of gene expression critical for successful implantation and placental development. The convergence of these findings across key signaling pathways, such as PI3K/AKT, IL-6/JAK/STAT, and MAPK/ERK, suggests that RPL represents a systems-level failure rather than a single molecular defect [9].

Translating these biomarkers into clinical settings has the potential to radically change diagnostics and therapeutics to achieve a successful pregnancy and provide information about the risks of delivering a baby with health complications.

## 3. Role of Functional Variants: Polymorphisms

Recent investigations into myeloperoxidase (MPO) polymorphisms have revealed compelling evidence for a genetic predisposition to hypertensive disorders of pregnancy [10]. The identification of rs2243828 and rs2071409 as functional polymorphisms that affect circulating MPO levels represents an important advancement in understanding the genetic architecture/background of gestational hypertension and preeclampsia. However, only a limited number of studies have explored the relationship between MPO polymorphisms and the risk of developing pre-eclampsia, making these studies particularly valuable.

The observation that the CC genotype of rs2243828 is associated with an increased risk of gestational hypertension, coupled with its strong effect on MPO concentration, provides a mechanistic understanding of how genetic variation translates into clinical phenotypes [11]. Particularly interesting is the protective effect of the ‘C, C’ haplotype in preeclampsia, suggesting a protective effect of reduced MPO levels for certain pregnancy complications. This finding challenges the inflammatory markers in pregnancy.

The study provides evidence that MPO genotypes and haplotypes influence circulating MPO levels in hypertensive disorders of pregnancy and may contribute to disease susceptibility, suggesting that MPO polymorphisms could serve as potential markers for identifying women at higher risk of developing hypertensive complications during pregnancy.

## 4. Placental Dysfunction as a Central Hub of Pregnancy Pathology

The demonstration that maternal GDM impairs fetoplacental insulin-induced vasodilation by disrupting the PI3K/AKT/eNOS pathway represents a paradigm shift in understanding fetal programming of cardiovascular disease [12]. The observed reduction in insulin receptor expression, coupled with deficient AKT phosphorylation in endothelial cells from GDM pregnancies, provides a mechanistic link between maternal metabolic dysfunction and offspring cardiovascular risk. A particularly prominent effect is the shift from NO-dependent to NO-independent vasodilation mechanisms in GDM vessels [13]. This suggests a fundamental endothelial reprogramming [14] that may persist postnatally/after delivery. The compensatory placental enlargement, along with its reduced efficiency, indicates an effort of adaptive response that ultimately fails to maintain optimal fetal–maternal exchange. This observation potentially contributes to the twice-increased cardiovascular disease risk in offspring exposed to GDM in utero.

Impaired maternal fetoplacental insulin-induced vasodilation may contribute to adverse neonatal outcomes observed in GDM pregnancies, such as earlier delivery, larger placenta, reduced placental efficiency, and the increased long-term risk of developing metabolic and cardiovascular diseases in the offspring [15].

## 5. Altered Transporter Expression in Maternal Obesity

The discovery that maternal obesity significantly reduces the placental expression of P glycoprotein (P-gp) has important implications for drug safety during pregnancy. Placental P-gp is a crucial efflux pump expressed in the trophoblast layer that functions as a transporter for drugs and toxins from the fetus, thereby acting as a protective barrier for the fetus.

The reduction in both ABCB1 mRNA and protein expression observed in obese mothers is particularly concerning, because approximately 10% of pregnancy medications are P-gp substrates [16]. This creates a hazardous situation for increased fetal exposure to potentially harmful substances [17].

Furthermore, the inhibition of P-gp through multiple substrates or inhibitors has been associated with congenital anomalies compared to monotherapy use. This alteration could potentially result in an increased risk of pregnancy complications and obesity-related drug contraindications linked to P-gp transport during pregnancy [18].

The validation of these findings across both rat models and human placental tissues strengthens the translational relevance. The correlation between reduced P-gp expression and adverse pregnancy outcomes, including reduced fetal weights, lower litter sizes, and decreased placental efficiency, suggests that transporter dysfunction may contribute to the broader pathophysiology of obesity-related pregnancy complications [19].

## 6. The Redox Balance Paradox

The dual nature of reactive oxygen species in placental biology presents a paradox that complicates therapeutic targeting. While controlled ROS levels are essential for normal placental functions, including trophoblast proliferation, differentiation, and angiogenesis, excessive oxidative stress drives multiple pregnancy complications [20]. The identification of specific biomarkers such as malondialdehyde, 8-isoprostane, protein carbonyls, and 8-hydroxy-2′-deoxyguanosine, provides a molecular fingerprint of oxidative damage that could enable early detection of pregnancies at risk [20]. The disappointing clinical outcomes from traditional antioxidant supplementation (with vitamins C and E) underscore the complexity of redox biology and the need for more sophisticated approaches, such as mitochondria-targeted antioxidants and activators.

In PE, inadequate placental perfusion leads to intermittent hypoxia/reoxygenation episodes, exacerbating oxidative stress (OS) and promoting the release of anti-angiogenic factors such as soluble fms-like tyrosine kinase-1 (sFlt-1) and soluble endoglin (sEng), which interfere with angiogenic signaling, further impairing placental function and contributing to maternal endothelial dysfunction [21]. Similarly, in IUGR, chronic hypoxia and OS can damage the placental vasculature and reduce nutrient and oxygen delivery to the fetus, leading to restricted growth.

## 7. Inter-Organ Communication Networks

One of the most radical findings is the demonstration of a placenta–liver–kidney axis in the pathogenesis of preeclampsia. An important discovery that placental secretions from PE-like conditions specifically promote hepatic serum amyloid A (SAA) production, which subsequently contributes to kidney inflammatory responses through TLR4-dependent mechanisms, reveals pregnancy complications as a systemic disorder involving inter-organ communication.

This concept of complicated organ network changes our understanding of PE from a primarily placental disorder to a multi-organ syndrome. The inability of SAA alone to fully reproduce the symptoms of PE suggests that additional liver-derived factors are involved and highlights the complexity of the communication pathways involved.

## 8. The Need for an Integrated Biomarker Panel

The number of identified biomarkers across multiple studies, from MPO polymorphisms to ITI-H4 in proteomics and from sFlt-1/PlGF ratios [21] to oxidative stress markers [22] presents both opportunities and challenges for clinical translation. The current limitation that 50–70% of RPL cases remain unexplained [23], despite extensive investigation, underscores the need for integrated biomarker panels rather than single markers [24].

The shift in our understanding of the placenta’s role, far from being merely a passive barrier, has revealed that it actively orchestrates maternal–fetal communication through sophisticated molecular signaling and immune and metabolic regulation. When these delicate processes are disrupted, whether by genetic polymorphisms, oxidative stress, metabolic dysfunction, or altered transporter expression, the consequences flow through multiple organ systems, creating a complex net of pathology.

## 9. Conclusions

The collective evidence from these important studies reveals maternal reproductive complications as complex multifactorial disorders requiring equally sophisticated approaches to diagnosis, prevention, and treatment.

The central role of the placenta underscores the need for in vivo methods to assess placental function; on the other hand, the identification of genetic polymorphisms that affect disease susceptibility opens avenues for risk stratification and preventive interventions in genetically predisposed individuals. Additionally, demonstrating inter-organ communication networks necessitates a shift to systems-level therapeutic strategies.

The vision of personalized medicine in maternal–fetal health, where genetic profiles, molecular biomarkers, and environmental factors inform individualized prevention and treatment strategies, is becoming increasingly achievable.
